# The Role of Tumor-Associated Neutrophils in Colorectal Cancer

**DOI:** 10.3390/ijms20030529

**Published:** 2019-01-27

**Authors:** Rei Mizuno, Kenji Kawada, Yoshiro Itatani, Ryotaro Ogawa, Yoshiyuki Kiyasu, Yoshiharu Sakai

**Affiliations:** Department of Surgery, Graduate School of Medicine, Kyoto University, Kyoto 606-8507, Japan; reimzn@kuhp.kyoto-u.ac.jp (R.M.); itatani@kuhp.kyoto-u.ac.jp (Y.I.); ogawaryo@kuhp.kyoto-u.ac.jp (R.O.); ykiyasu@kuhp.kyoto-u.ac.jp (Y.K.); ysakai@kuhp.kyoto-u.ac.jp (Y.S.)

**Keywords:** neutrophils, colon cancer, tumor microenvironment, cancer immunity

## Abstract

Colorectal cancer (CRC) is one of the most common causes of cancer deaths worldwide and the number of CRC patients is increasing progressively. Despite the improvement of the surgical techniques and chemotherapy, we have not completely overcome this disease yet due to the metastases. Therefore, understanding the mechanisms through which metastasis occurs is important for overcoming CRC. Normal host cells in the tumor microenvironment, such as macrophages and fibroblasts, have been reported to promote the growth of CRCs. Although neutrophils were originally considered to have defensive functions against tumor cells, it has been revealed that some populations of neutrophils, called as tumor-associated neutrophils (TANs), have tumor-supportive functions. The plasticity between tumor-suppressive and -supportive neutrophils are regulated by transforming growth factor (TGF)-β and Interferon-β signaling. Some studies have demonstrated that TANs promote the spread of cancer cells to distant organs. TANs contribute to the tumor invasion and angiogenesis through the production of matrix metalloproteinase-9 (MMP9), vascular endothelial growth factor (VEGF), and hepatocyte growth factor (HGF) in the primary and metastatic sites. Neutrophils also promotes tumor cell dissemination by capturing circulating tumor cells using neutrophil extracellular traps and promote their migration to distant sites. The neutrophil-to-lymphocyte ratio is a well-defined predictive marker for CRC patients. In this review, we highlight the molecular signaling between TANs and CRC cells and the possibility of TANs as a potential target for cancer therapy.

## 1. Introduction

Colorectal cancer (CRC) is one of the most common causes of cancer-related deaths worldwide [1,2,3]. Despite advances in surgical techniques, chemo-drugs, and molecular-targeted drugs (e.g., bevacizumab and cetuximab targeting vascular endothelial growth factor (VEGF) and epidermal growth factor receptor (EGFR), respectively) [4], the number of CRC patients is increasing progressively [5,6]. At least one third of CRC patients develop liver metastases, and CRC-related death is usually attributable to distant metastasis [7,8]. Once the disease spreads to distant organs, neither conventional chemotherapy nor current targeted therapy offers significant benefits. Therefore, it is important to understand the mechanisms through which metastasis occurs and to find therapeutic targets for distant metastasis.

**Figure 1:**
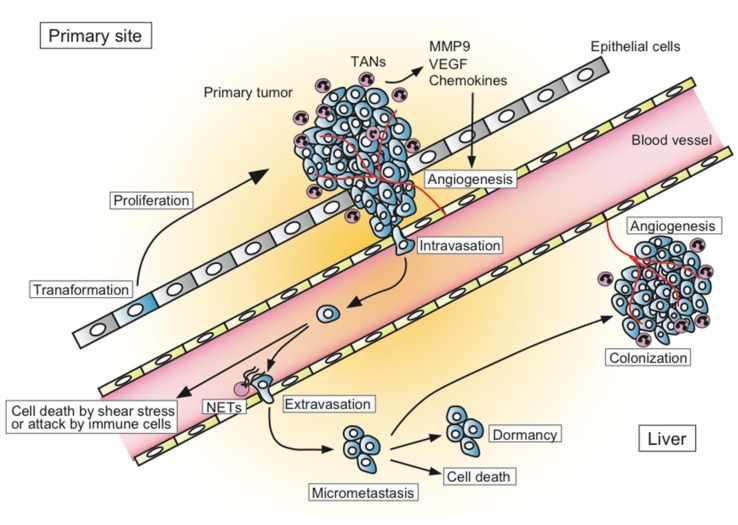
Overview of the process of liver metastasis.

The process of metastatic formation can be divided into several successive steps (Figure 1). In the primary tumor site, the transformed tumor cells begin to grow and secrete angiogenic factors, which results in extensive vascularization. Tumor cells locally invade through the activation of proteases and intravasate into thin-walled vessels (i.e., venules and lymphatic vessels) and enter the blood circulation. Embolization of single cancer cell or aggregates occur next. During this process, most circulating cancer cells are destroyed by the shear forces of blood flow or by the attack from components of the host immune system such as natural killer cells. If the tumor cells can survive in blood circulation, they become trapped in the capillary beds of distant organs. Finally, tumor cells extravasate into the organ parenchyma and start to form micrometastases. Some tumor cells within micrometastatic sites die due to the attack of host immune cells, while others survive in a dormant state that exits from the cell cycle and balances their proliferation and apoptosis. Although less is understood about how dormancy is broken, some tumor cells start to proliferate and expand through the secretion of angiogenic factors and the activation of proteases to form metastatic colonies. Only a limited number of cancer cells can form metastases in distant organs [9,10]. The transition from pre-angiogenic to angiogenic metastasis is a rate-limiting step in the occurrence of liver metastasis, which suggests that the development of an angiogenic phenotype is a key step for metastatic progression [11].

However, the precise underlying mechanisms by which cancer cells survive in the hostile environment and develop metastatic sites still remain unclear. It has been reported that several types of host cells, such as fibroblasts (cancer-associated fibroblasts: CAF), macrophages (tumor-associated macrophages: TAMs), and mesenchymal stem cells, play important roles in the formation of the tumor microenvironment [12,13,14]. In addition, recent accumulating evidence has shown that some populations of neutrophils, known as tumor-associated neutrophils (TANs), could support the growth, invasion, and angiogenesis of cancer cells, although they have been classically considered to exhibit a defensive response against tumor cells. They have also been reported to exert supportive functions in the development of metastasis. Here, we highlight the role of TANs in supporting the development of distant CRC metastasis, especially liver metastasis.

Liver metastasis is a complex, multistep process. In the primary tumor site, transformed tumor cells start to proliferate and secrete angiogenic factors, which results in extensive vascularization. Tumor cells locally invade blood vessels. Most circulating tumor cells are destroyed by the shear forces of blood flow or by the attack from the host immune system such as natural killer cells. If the tumor cells can survive in blood circulation, they become trapped in the capillary beds of distant organs. Finally, tumor cells extravasate into the organ parenchyma and start to form micrometastases. Some tumor cells die and others survive in a dormant state. Some tumor cells break the dormancy and start to proliferate and expand through the secretion of angiogenic factors and the activation of proteases.

## 2. Tumor-Associated Neutrophils (TANs)

Normal host cells in the tumor microenvironment, such as CAFs and TAMs, assist in the growth, invasion, and metastasis of cancer cells [13,14]. It has become evident that bone marrow-derived cells including TAMs, TANs, and myeloid-derived suppressor cells (MDSCs), contribute to tumor progression [12,15,16,17,18,19]. Recently, a number of studies have demonstrated through immunohistochemical analyses that neutrophils, which are another leukocyte population, were intermingled in various cancer tissues. Chemokines are small peptides binding to G protein-coupled receptors to induce chemoattraction, inflammation, and/or angiogenesis [20]. They are one of the key factors that facilitate cancer metastasis [21]. Tumor cells often produce several inflammatory chemokines, including neutrophil-attracting CXC-chemokines [22,23]. The migration of neutrophils toward the tumor is mainly mediated by CXC-chemokines that bind to CXCR1 and/or CXCR2 [24,25,26].

Neutrophils have been originally viewed as the first-responder of the innate immune system in the resistance against extracellular pathogens. However, recent evidence has added a new aspect on the function of neutrophils. Neutrophils are involved in the regulation of innate and adaptive immune systems, and can be polarized towards distinct phenotypes in response to environmental signals [22]. They are classically characterized based on their ability to induce phagocytosis, release lytic enzymes, and produce reactive oxygen species (ROS) [27,28]. In the context of the tumor microenvironment, accumulating evidence has revealed the prominent role of neutrophils in infiltrating tumor tissues to promote their growth, invasion, angiogenesis, and metastasis in various types of cancers, although they were initially considered to have a defensive function against tumor cells [29,30,31].

TAMs are divided into two populations: the anti-tumorigenic “M1” phenotype and pro-tumorigenic “M2” phenotype. As with TAMs, recent studies have suggested that TANs also exhibit considerable plasticity and are capable of polarization into either an anti-tumorigenic “N1” phenotype or a pro-tumorigenic “N2” phenotype [22,32,33]. Their surface markers, transcriptional regulators, and cytokine profiles remain to be investigated. Neutrophils are known to secrete several inflammatory, immunoregulatory, and angiogenic factors, including neutrophil elastase [34], matrix metalloproteinases (MMPs), vascular endothelial growth factor (VEGF) [35,36], and hepatocyte growth factor [37], which can exhibit paracrine effects on the tumor microenvironment. “N1” neutrophils exhibit increased cytotoxicity and reduced immunosuppressive ability by the production of tumor necrosis factor (TNF)-α, intercellular adhesion molecule (ICAM)-1, ROS, and Fas and by decreasing arginase expression. In contrast, “N2” neutrophils support tumor expansion by expressing arginase, MMP-9, VEGF, and numerous chemokines including CCL2, CCL5 and CXCL4 [32]. Fridlender et al. reported that transforming growth factor (TGF)-β signaling functions as a regulator between the N1 and N2 phenotypes. TGF-β within tumors skews differentiation toward the N2 phenotype, while inhibition of TGF-β signaling induces an anti-tumoral N1 phenotype [32]. Interferon-β was also recently reported to induce an N1 phenotype [38]. Taken together, the phenotype of TANs depends on the signals encountered in the tumor microenvironment.

Moreover, some studies have recently investigated the possible involvement of neutrophil extracellular traps (NETs) in promoting the migration and extravasation of cancer cells. NETs are composed mainly of fibers of decondensed DNA, and are decorated with proteins released from activated neutrophils [39,40,41,42,43]. They act as meshes that trap microorganisms and, in turn, promote the interaction between pathogens and neutrophil-derived effector molecules [39,44]. NETs have recently been suggested to capture circulating cancer cells and promote their migration to new sites [45,46]. NETs has also been shown that they can activate toll-like receptor 9 on CRC cells, resulting in cellular growth, migration, and invasion via activation of mitogen-activated protein kinase (MAPK) signaling [47]. Najmeh et al. recently reported the importance of β1-integrin expression on both circulating cancer cells and NETs in mediating cancer cell adhesion to NETs in vivo, resulting in the development of metastatic diseases [48].

In addition, TAMs and TANs have the potential to drive tumor angiogenesis. In various murine models, TAMs and TANs are major sources of MMP9 [49], which promotes angiogenesis by its extracellular matrix-degrading properties [50]. In a genetically-engineered mouse model of pancreatic cancer, MMP9 expression was exclusively found in neutrophils, and neutrophil depletion inhibited the angiogenic switch in the primary tumors [51]. Moreover, in a tumor xenograft model, granulocyte colony-stimulating factor (G-CSF)-induced upregulation of Bv8 (known as prokineticin-2) in neutrophil was shown to promote tumor angiogenesis [52]. G-CSF facilitated neutrophil recruitment into the tumor, stimulated Bv8 expression, and promoted angiogenesis, which resulted in resistance against anti-VEGF treatment [53,54,55].

CXCR2 and its ligands (i.e., CXCL1, CXCL2, CXCL3, CXCL5, CXCL7, and CXCL8) are responsible for the recruitment of neutrophils under normal physiological conditions and are implicated in the mobilization of TANs [56]. In tumor-bearing mouse models, targeting CXCR2-mediated TAN mobilization has been reported to increase the number of tumor-infiltrating lymphocytes and potentiate anti-programmed death 1 checkpoint blockade [57,58,59,60]. Nywening et al. have recently reported that dual blocking of CXCR2^+^ TANs and CCR2^+^ TAMs disrupted the recruitment of myeloid cells and improved anti-tumor immunity in a mouse model of pancreatic cancer [61]. Recently, we have found that loss of SMAD4 from CRC cells results in the secretion of CXCL1 and CXCL8 to recruit CXCR2^+^ neutrophils, and that, in turn, the recruited neutrophils abundantly produce CXCL1 and CXCL8, which further prompts the accumulation of CXCR2^+^ neutrophils and results in an amplification of the cytokine/chemokine milieu shaped by the CXCL1/8-CXCR2 axis [62].

## 3. TANs in Human CRC

The relationship between TAN infiltration and human cancer prognosis has not been systemically investigated, although some studies have reported the role of neutrophils as a prognostic factor in various types of human cancers. The increase of neutrophil count in peripheral blood (i.e., neutrophil-to-lymphocyte ratio (NLR)) has been shown to be related to poor clinical outcomes in pancreatic cancer, gastric cancer, and breast cancer [63,64,65], emphasizing the importance of neutrophils in cancer biology. High NLR has also been demonstrated as a poor prognostic factor in CRC patients. Li et al. retrospectively analyzed a cohort of 354 CRC patients with stage I–III cancer and revealed a strong relationship between dynamic changes in NLR and overall survival [66]. Other studies have also reported that high NLR had an adverse effect on overall survival in CRC patients subjected to curative surgery [67,68]. High NLR was also shown to predict poor outcome following hepatic resection for liver metastasis of CRC [69]. Dell’Aquila et al. showed that high NLR was also a poor prognostic factor in unresectable metastatic CRC patients treated with bevacizumab plus chemotherapy [70].

However, the effect of intratumoral neutrophils on the survival for CRC patients is still unclear. Rao et al. demonstrated that the increase in intratumoral neutrophils was associated with malignant phenotypes and could predict adverse prognosis in CRC [71]. On the other hand, Berry et al. analyzed the number of neutrophils in CRC tissues by counting neutrophils manually based on their morphology because of the lack of neutrophil-specific antibodies and demonstrated that high levels of TANs were associated with improved overall survival in patients with stage II CRC [72]. As described, the signals from the tumor microenvironment that determine the N1 and N2 neutrophil phenotypes might affect the results. We have recently reported that loss of SMAD4-promoted CCL15 expression from CRC cells to recruit CCR1^+^ myeloid cells through the CCL15-CCR1 axis, and that CCL15 expression in primary and metastatic CRCs was a predictor of CRC patients’ prognosis [73,74,75]. Most CCR1^+^ myeloid cells recruited into the primary CRC and metastatic CRC were of the granulocytic-MDSC phenotype (CD11b^+^, CD33^+^, HLA-DR^−^, and CD15^+^) [74] and TAN phenotype (CD11b^+^, CD33^−^, HLA-DR^−^, CD15^+^, and CD16^+^) [75], respectively. MDSCs constitute a heterogeneous population of immature myeloid cells at various differentiation stages and represent a group of myeloid cells that suppress immune responses. Because TANs and MDSCs share a common set of markers and are morphologically similar, it remains unclear whether TANs and MDSCs are separate populations or not [29,76,77]. In addition, MDSCs were reported to differentiate into mature TAMs or TANs within the tumor [64]. Further analysis is needed to evaluate the significance of intratumoral neutrophils in CRC.

## 4. TANs in Animal Model for CRC Liver Metastasis 

A number of studies have tried to clarify the underlying mechanisms by which neutrophils support liver metastasis of CRC cells in vitro and in vivo using metastatic mouse models (Table 1).

In a mouse model of liver metastasis in which tumor cells were inoculated through splenic injection, Hirai et al. demonstrated the interaction between neutrophils and CRC cells during the process of colonization within the liver [78]. They found that mouse CCL9 (mCCL9)-expressing CRC cells recruited myeloid cells expressing CCR1, the cognate receptor of mCCL9, to form early metastatic foci in the liver, and that four distinct types of myeloid cells were recruited to the site of liver metastasis: CCR1^+^ neutrophils, monocytes, eosinophils, and fibrocytes. CCR1^+^ neutrophils produce MMP9, which helps cancer foci to expand in the early stage, and trigger the recruitment of fibrocytes and monocytes that produce MMP2 during the later stages. We have also reported that the recruitment of CCR1^+^ myeloid cells facilitates primary CRC invasion [74] and metastasis to the liver [73] and lungs [75]. Using *Ccr1*-knockout mice, Rodero et al. reported that CCR1 expression in hematopoietic and non-hematopoietic cells facilitated liver metastasis through myeloid cell accumulation in the metastatic tumors [79]. Moreover, using the bone-marrow transplantation model of *Ccr1*-knockout mice, we have recently found that knockout of CCR1 expression in myeloid cells significantly suppressed tumor growth in primary and metastatic CRCs [80], which suggests that the use of CCR1 inhibitors can be a promising strategy to treat CRC. 

CXCL8, or interleukin(IL)-8, is the first-described angiogenic chemokine, and is secreted from CRC cells stimulated by TNF-α and IL-1α [88]. CXCL8 is also induced by hypoxia as a hypoxia-inducible factor 1-independent pathway to preserve tumor angiogenesis as a compensatory pathway of VEGF [89]. Kumar et al. revealed that the upregulation of CXCL8 secreted from CRC cells promoted CRC liver metastasis [81]. They demonstrated that the expression of CXCL8 was upregulated in the invasion front of the tumor and that shRNA-mediated knockdown of CXCL8 resulted in significantly decreased cell proliferation, migration, and invasion in vitro and dramatic reduction of tumor metastasis in vivo. They also revealed that the knockdown of CXCL8 resulted in a reduction in VEGF-A expression, suggesting that overexpression of CXCL8 could induce a VEGF-dependent angiogenic response. CXCL8 could also promote angiogenesis in a VEGF-independent manner in CRC [90]. Using a CRC xenograft model, Yamamoto et al. reported that the CXCL1-CXCR2 axis was important in modulating the pre-metastatic niche of CRC liver metastasis, and that TSU68 (an inhibitor of VEGF receptor 2, platelet-derived growth factor receptor β and fibroblast growth factor (FGF) receptor 1) suppressed CXCL1 expression in the pre-metastatic liver, resulting in suppression of CXCR2^+^ neutrophils homing and subsequent liver metastasis [82]. In *Cxcr2-*knockout mice, the deficiency of the CXCL8-CXCR2 axis in the host cells resulted in the inhibition of CRC growth and metastasis [91]. Moreover, the CXCR1/CXCR2 antagonist inhibited CRC liver metastasis by decreasing tumor angiogenesis and facilitating tumor cell apoptosis in a mouse model [83]. Combination of a CXCR2 antagonist and oxaliplatin was reported to result in a great decrease of tumor growth and angiogenesis in xenograft models [92]. Using CXCL8-expressing transgenic mice, Asfaha et al. reported that CXCL8 expression increased the mobilization of immature myeloid cells in dextran sodium sulfate-induced colitis, which exacerbated acute inflammation and accelerated colon carcinogenesis [93].

Other signaling molecules are indicated to be involved in the pro-tumorigenic function of neutrophils. Gordon-Weeks et al. demonstrated that human CRC metastasis to the liver and experimental murine models of liver metastasis were infiltrated by neutrophils. They showed that metastasis-associated neutrophils in the liver substantially expressed FGF 2, a pro-angiogenic molecule, indicating neutrophil polarization by the tumor microenvironment [84]. Of note, neutralizing anti-FGF2 antibodies could cause neutrophil depletion and reduce liver metastatic colony growth and vascular density.

Some studies have revealed that signaling pathways in the liver itself make it more susceptible to metastasis and regulate the polarization of the TAN phenotype. In transgenic mice with a conditional, liver-specific insulin-like growth factor (IGF)-1 deficiency, Rayes et al. showed that IGF-1 signaling in the liver was important in the polarization of neutrophils. In mice subjected to IGF-1 deficiency three weeks but not two days prior to the inoculation of CRC cells, infiltrated neutrophils in the liver did not show characteristics of tumor-promoting phenotypes, although the number of neutrophils was increased. They suggested that sustained IGF-1 deficiency is necessary to alter the neutrophil phenotype [85].

Seubert et al. demonstrated that high systemic expression of tissue inhibitor of metalloproteases-1 (TIMP-1) increased liver susceptibility towards metastasis by triggering the formation of a pre-metastatic niche. High systemic levels of TIMP-1 resulted in increased hepatic CXCL12 levels, which in turn promoted recruitment of neutrophils to the liver. Both inhibition of CXCL12-dependent neutrophil recruitment and systemic depletion of neutrophils could suppress TIMP-1-induced susceptibility towards liver metastasis [86]. 

Using a colitis-associated CRC mouse model, Ma et al. reported that prostaglandin E2 receptor subtype EP2 was expressed in infiltrating TANs and CAFs in CRC, and that the expression of cytokines such as TNF-α, IL-6, CXCL1, cyclooygenase-2, and Wnt5A was amplified in tumor lesions via EP2 expression in TANs and CAFs. Importantly, treatment with a selective EP2 antagonist potently suppressed tumor growth in this model [87].

## 5. Limitations of Studies on the Interaction Between Neutrophils and Cancer Cells

In most studies, the interaction between neutrophils and CRC cells has been analyzed in vitro using cancer cells, isolated neutrophils and human umbilical vein endothelial cells. However, isolated neutrophils for in vitro experiments do not behave normally as they are primed or pre-activated during the process of isolation [94]. In addition, some studies with in vivo experiments use xenograft models to show the interaction between neutrophils and CRC cells. However, xenograft models are less reliable for cancer metastasis. Therefore, we must keep in mind that the mechanisms that are observed through in vitro experiments or in vivo experiments with xenografts do not always represent actual in vivo biological phenomena. Although the development of microscopic techniques, such as confocal microscopy and two-photon excitation microscopy, combined with fluorescent proteins have enabled us to visualize various biological events in vivo, it is still challenging to observe the interaction between cancer cells and neutrophils during the process of liver metastasis. Further in vivo studies will be required.

Accumulating evidence indicated that exogenous and endogenous factors, such as diet, alcohol, smoking, obesity, lifestyle, environmental exposures, and microbiome, can influence the tumor–immune interactions. Through recruitment of host immune cells, the gut microbiome could generate a proinflammatory microenvironment that is conductive for CRC progression [95]. Molecular pathological epidemiology (MPE) integrates tumor immunology into population health sciences, and links the exposures and germline genetics to tumor and immune characteristics using bioinformatics, in vivo pathology and omics technologies. This kind of integrative approach would be important to understand the mechanisms of tumor progression, effective prevention and therapeutic strategies for precision medicine for CRC [96,97].

## 6. TANs as a Potential Target for Cancer Therapy

The evaluation of TANs as a potential therapeutic target is still ongoing because their role in cancer development and metastasis is not completely understood. Considering the role of TANs in tumor progression, targeting neutrophils in cancer could be a potential new anti-tumor therapy. However, depletion of neutrophils in humans could lead to self-defeating immunosuppression as neutrophils are essential for host defense against infection. Therefore, it has been postulated that blocking specific populations of neutrophils, especially TANs, can be beneficial and promote tumor regression or metastatic spread. 

Since TGF-β modulates the pro- and anti-tumor phenotypes of neutrophils, TGF-β blocking could theoretically be a potential therapeutic strategy. Multiple trials that have tested the effect of TGF-β blocking failed as a result of significant side effects because TGF-β is involved in numerous physiological pathways [98]. New strategies and molecules directed toward either TGF-β or its receptors are currently being clinically tested [99]. In CRC, a TGF-β receptor II antibody (IMC-TR1, also known as LY3022859) has been developed, and the murinized derivative exhibited good response in mouse models of CRC and breast cancer [100]. At the time of writing, this drug is in a phase I trial for patients with advanced solid tumors including CRC for whom standard therapies have failed (NCT01646203) [101].

Chemokine blocking could be another effective strategy resulting in impaired neutrophil recruitment to the tumor. Since CXCL8 secreted from CRC cells recruits neutrophils [81,88,89], blocking the CXCL8 axis by neutralizing antibodies could be a good therapeutic approach. However, its consequences on the phenotype of circulating or intratumoral neutrophils in human cancer are still unknown. Therefore, further studies are needed to gain a detailed understanding of TANs and CRC and for the application of future novel anti-tumor therapies.

## 7. Conclusions

Accumulating evidence has shown that neutrophils infiltrating CRC tissues, as well as macrophages and fibroblasts, play important roles in the tumor microenvironment. TANs exhibit the plasticity between the anti-tumorigenic N1 or tumor-promoting N2 phenotype, which is determined by signals from surrounding tissues. In this review, we highlighted the role of neutrophils in promoting liver metastasis of CRC. NLR is a well-defined predictive biomarker for CRC patients. Studies with animal models for liver metastasis of CRC demonstrated the underlying mechanisms by which neutrophils promoted liver metastasis, which could contribute to novel therapeutic targets and biological markers.

## Figures and Tables

**Table 1:** TANs in animal models for CRC metastasis.

Authors	Reference	Animal	Cell	Molecules	Effect
Hirai et al.	[78]	Mouse	CRC cells	CCL-9	CCL-9 in CRC cells recruit CCR1^+^ neutrophils which produce MMP9 for cancer expansion
Rodero et al.	[79]	Mouse	Hematopoietic/nonhematopoietic cells	CCR1	CCR1 expression by both hematopoietic and non-hematopoietic cells favors tumor aggressiveness
Kiyasu, Y.	[80]	Mouse	Myeloid cells	CCR1	*CCR1*-knockout in myeloid cells suppress CRC liver metastasis
Kumar et al.	[81]	Mouse	CRC cells	CXCL8	CXCL8 promotes neutrophil recruitment, metastasis, angiogenesis and invasion
Yamamoto et al.	[82]	Mouse		CXCL1/CXCR2	CXCL1/CXCR2 axis is important in cancer metastasis
Varney et al.	[83]	Mouse	Systemic	CXCR1/CXCR2	Systemic inhibition of CXCR1/CXCR2 induced apoptosis and inhibited angiogenesis in the liver metastasis
Gordon-Weeks et al.	[84]	Mouse	TANs	FGF2	FGF2 in TANs induce polarization of neutrophils
Rayes et al.	[85]	Mouse	Liver	IGF-1	Sustained IGF-1 deficiency in liver alters the neutrophil phenotypes
Seubert et al.	[86]	Mouse	Systemic	TIMP-1	Systemic TIMP-1 expression promotes neutrophil recruitment through the increase of hepatic SDF-1 and increase the liver susceptibility
Ma et al.	[87]	Mouse	TANs	EP2	EP2 signaling in TANs promotes tumor growth through TNF-β, IL-6, CXCL1, COX-2, and Wnt5A

## Abbreviations

CAF	cancer-associated fibroblasts
CRC	colorectal cancer
EGFR	epidermal growth factor receptor
FGF	fibroblast growth factor
G-CSF	granulocyte colony-stimulating factor
ICAM	intercellular adhesion molecule
IGF	insulin-like growth factor
IL	interleukin
MAPK	mitogen-activated protein kinase
MDSC	myeloid-derived suppressor cell
MMP	matrix metalloproteinase
MPE	molecular pathological epidemiology
NETs	neutrophil extracellular traps
NLR	neutrophil-to-lymphocyte ratio
ROS	reactive oxygen species
TAM	tumor-associated macrophage
TAN	tumor-associated neutrophil
TGF-β	transforming growth factor-beta
TIMP-1	tissue inhibitor of metalloproteases-1
TNF	tumor necrosis factor
VEGF	vascular endothelial growth factor
